# Systemic lupus erythematosus–accelerated atherosclerosis: mechanistic insights and clinical implications

**DOI:** 10.3389/fimmu.2026.1818480

**Published:** 2026-04-22

**Authors:** Dexiu Li, Dongyue Ma, Liying Zheng, Lin Zhao, Ziyi Qiu, Mei Xue

**Affiliations:** 1 Xiyuan Hospital, China Academy of Chinese Medical Sciences, Beijing, China; 2 National Clinical Research Center for Chinese Medicine Cardiology, Beijing, China; 3 Department of Andrology, Xiyuan Hospital of Clinical Medical College of Beijing University of Chinese Medicine, Beijing, China

**Keywords:** autoimmune disease, coronary atherosclerosis, IFN-I, immune-inflammatory mechanisms, systemic lupus erythematosus

## Abstract

Premature coronary atherosclerosis remains a primary driver of late-stage mortality in systemic lupus erythematosus (SLE), independent of traditional cardiovascular risk profiles. This mini-review outlines the multifaceted immunometabolic pathways that underpin accelerated atherogenesis in SLE patients. We examine how chronic systemic inflammation modifies the lipoprotein profile toward a pro-oxidant state, characterized by dysfunctional HDL and elevated oxidized LDL. Central to this vascular pathology are type I interferon-driven cascades, excessive neutrophil extracellular trap release, and biased macrophage polarization toward pro-atherogenic phenotypes. Furthermore, the roles of pathogenic autoantibodies, genetic susceptibility, and the metabolic impact of specific immunosuppressants are explored. Integrating these mechanistic insights is essential for refining cardiovascular risk assessment and identifying novel immunomodulatory interventions. Ultimately, understanding the unique SLE–atherosclerosis axis provides a foundation for reducing cardiovascular morbidity and improving long-term outcomes in this vulnerable population.

## 1 Introduction

Coronary atherosclerosis (CAS), the primary pathological basis of coronary artery disease, is characterized by lipid deposition in the arterial intima, chronic inflammation, and the formation of fibrous plaques, ultimately leading to myocardial ischemia, myocardial infarction, or even sudden cardiac death. Atherosclerotic cardiovascular disease remains the leading cause of mortality and disability worldwide. Its incidence continues to rise globally, posing a major global health challenge (1). Systemic lupus erythematosus (SLE) is a multisystem autoimmune disorder characterized by the sustained production of autoantibodies and immune complex deposition, leading to diverse and complex clinical manifestations (2). Improvements in immunosuppressive regimens and supportive care have markedly enhanced long-term survival among SLE patients. However, as early mortality due to active disease has declined, cardiovascular disease has emerged as a leading cause of late mortality in this population (3).

Epidemiological studies consistently demonstrate that patients with SLE carry a markedly elevated risk of premature coronary atherosclerosis compared with age- and sex-matched controls, particularly among young and premenopausal women who would otherwise be considered at low cardiovascular risk (4). Traditional cardiovascular risk factors, such as dyslipidemia, hypertension, smoking, and diabetes, fail to fully account for this disproportionate risk. Additionally, traditional preventive strategies focused on lipid lowering have yielded disappointing results in this population. Large-scale clinical trials, such as the Lupus Atherosclerosis Prevention Study (LAPS) and the Atherosclerosis Prevention in Pediatric Lupus Erythematosus (APPLE) trial, failed to demonstrate a significant benefit of statins in slowing the progression of subclinical atherosclerosis in SLE patients (5, 6). The LAPS substudy further supports these clinical observations, revealing that statin therapy does not normalize the pro-atherogenic cholesterol transport gene expression profile in macrophages exposed to SLE patient plasma, thus failing to correct the underlying atherogenic dysregulation induced by lupus (7). These negative outcomes underscore that atherosclerosis in SLE is not merely a metabolic byproduct but a distinct immunopathological process. Therefore, identifying the “residual” immune-driven risk, which persists despite lipid control, is essential for developing targeted interventions.

Growing evidence supports the concept that SLE and atherosclerosis share overlapping immunopathogenic pathways, including chronic systemic inflammation, oxidative stress, complement activation, endothelial injury, neutrophil extracellular traps (NETs) formation, and aberrant T- and B-cell responses (8–10). Immune complex deposition and type I interferon (IFN-I) signaling further amplify vascular inflammation and promote plaque instability (11). In this context, SLE may serve as a human model of immune-driven accelerated atherosclerosis. Understanding the mechanistic links between SLE and coronary atherosclerosis is of substantial clinical importance. First, it may facilitate earlier cardiovascular risk stratification in SLE patients. Second, it provides insight into potential therapeutic targets that extend beyond conventional lipid-lowering strategies. Finally, dissecting lupus-associated atherogenesis may also shed light on the broader immunoinflammatory basis of atherosclerosis in the general population.

Therefore, this mini review aims to summarize current evidence regarding the pathophysiological mechanisms by which SLE promotes coronary atherosclerosis, with particular emphasis on immune dysregulation, endothelial dysfunction, and inflammation-driven vascular remodeling. By integrating experimental and clinical findings, we seek to clarify the SLE–atherosclerosis nexus and highlight potential directions for future research and therapeutic intervention.

## 2 Pathophysiological mechanisms linking SLE to accelerated atherosclerosis

### 2.1 HDL dysfunction and susceptibility to LDL oxidation

In SLE, lipoprotein abnormalities a extend beyond alterations in conventional lipid profiles to include profound functional and structural disturbances in HDL and LDL. The chronic inflammatory state, enhanced oxidative stress, and immune-mediated modifications characteristic of SLE collectively drive the transformation of HDL from a protective molecule with anti-inflammatory and antioxidant properties into a pro-inflammatory entity, often termed dysfunctional HDL (12). Elevated levels of myeloperoxidase (MPO) and reduced paraoxonase-1 (PON1) activity in these patients accelerate the oxidative modification of apolipoprotein A1 (ApoA1), compromising reverse cholesterol transport and diminishing the anti-atherosclerotic functions of HDL (13). Concurrently, increased levels of oxidized HDL bind to lectin-like oxidized LDL receptor 1(LOX-1), impeding signal transducer and activator of transcription activator 3 activation. This results in upregulation of Toll-like receptors (TLRs) and enhanced synthesis of pro-inflammatory cytokines. Compared with the general population, SLE patients have lower HDL levels, smaller HDL particle size, reduced ApoA1 content, elevated oxidation, and higher serum amyloid A levels. These alterations confer a pro-inflammatory phenotype on HDL, which promotes monocyte recruitment and vascular inflammatory responses, thereby attenuating its atheroprotective effects (12, 14).

In parallel with HDL dysfunction and impaired PON1 activity, SLE patients demonstrate heightened susceptibility to LDL oxidation and significantly elevated circulating levels of oxidized LDL (ox-LDL) (15). ox-LDL markedly enhances the expression of endothelial adhesion molecules, stimulates lipid uptake by macrophages leading to foam cells formation, and impairs macrophage capacity to clear apoptotic cells—culminating in expansion of the necrotic core and increased plaque instability. Furthermore, Clinical studies suggest that SLE is associated with elevated levels of highly negatively charged LDL subfractions, which upregulate the expression of vascular cell adhesion molecule-1 (VCAM-1), monocyte chemoattractant protein-1 (MCP-1), and matrix metalloproteinase-2 (MMP2). These subfractions possess stronger pro-inflammatory and chemotactic capabilities, exacerbating endothelial injury and vascular inflammation, and significantly increasing atherosclerosis risk in SLE patients (16). Proprotein convertase subtilisin/kexin type 9 (PCSK9) can reduce LDL degradation, promote platelet activation, and drive the pro-inflammatory processes of monocytes and macrophages (17). Elevated PCSK9 levels in an observational study of SLE patients promote LDL receptor degradation, increasing the accumulation of circulating LDL, while simultaneously amplifying vascular inflammation and coronary atherosclerosis risk (18). In SLE, the loss of HDL-mediated protection coupled with enhanced pro-atherogenic LDL effects creates a pathological lipoprotein profile skewed toward a pro-inflammatory and pro-oxidant state. This lipid milieu predisposes SLE patients to premature or accelerated atherosclerosis at younger ages, even in the absence of typical cardiovascular risk factors. Processes such as vascular remodeling, intimal thickening, and plaque formation occur more rapidly in these individuals compared with the general population.

### 2.2 Immune-inflammatory mechanisms

#### 2.2.1 IFN-I

A sustained elevation of IFN-I is a hallmark immunological feature of SLE and is closely associated with disease activity (10). IFN-I production is primarily triggered by innate immune sensors, including TLRs, RIG-I–like receptors (RLRs), and the cGAS–STING pathway. Chronic activation of these contributes to broad immune dysregulation and a heightened inflammatory state. IFN-I downregulates endothelial nitric oxide synthase (eNOS) expression, reducing nitric oxide (NO) bioavailability and impairing endothelium-dependent vasodilation. Consistent with this, flow-mediated vasodilation was attenuated in SLE patients compared with healthy controls (19) Furthermore, elevated IFN-I levels activate the JAK–STAT signaling pathway, which suppresses IL-1β and vascular endothelial growth factor A (VEGF-A), induces apoptosis of endothelial progenitor cells (EPCs) and circulating angiogenic cells, and promotes the differentiation of myeloid precursors toward non-angiogenic phenotypes. These effects collectively deplete the pool of cells capable of differentiating into mature endothelial cells (20, 21). IFN-I also downregulates monocyte-derived angiopoietin-1 while increasing circulating angiopoietin-2 levels and promoting TIE1 cleavage, thereby disrupting TIE2 receptor phosphorylation. These changes lead to decreased expression of endothelial barrier–related genes and increased endothelial vulnerability (22).

Within the context of atherogenesis, IFN-I exacerbates vascular inflammation and promotes foam cell formation by inducing interferon-stimulated genes, driving macrophage polarization to the pro-inflammatory M1 phenotype, and stimulating inflammatory cytokines secretion (20). It additionally upregulates scavenger receptor A (SR-A) on macrophages, facilitating ox-LDL uptake and accelerating foam cell formation. IFN-I also increases endothelial expression of adhesion molecules such as VCAM-1 and intercellular adhesion molecule-1 (ICAM-1), and MCP-1, thereby promoting leukocyte adhesion and transmigration and intensifying vascular inflammation (23). Evidence also indicates that IFN-I elevates levels of circulating smooth muscle progenitor cells and maintains them in an immature state, contributing to medial layer remodeling and atherogenesis (24). Overall, IFN-I–mediated immune dysregulation and endothelial dysfunction converge at multiple stages of SLE-associated atherogenesis—including impaired endothelial repair, amplified inflammation, dysregulated lipid metabolism, and maladaptive vascular remodeling—ultimately fostering a pro-atherogenic microenvironment that perpetuates disease progression.

#### 2.2.2 Neutrophils

NETs are decondensed chromatin networks comprising DNA, histones, and granule proteins that neutrophils expel to combat infectious or inflammatory threats. In patients with SLE, circulating NET-specific markers, such as MPO–DNA complexes, are markedly elevated (25). NET formation is not only more readily triggered by autoantibodies and immune complexes, but their clearance is also impaired due to decreased DNase I activity, resulting in their persistent accumulation *in vivo* (26). Excessive NETs, via their DNA–protein complexes and oxidized constituents, activate plasmacytoid dendritic cells and amplify TLR and STING-dependent signaling, thereby enhancing IFN-I production (27). NETs also stimulate NLRP3 inflammasome activation in macrophages, leading to IL-1β and IL-18 secretion, and contain abundant ROS-generating enzymes that contribute to marked local oxidative stress (28, 29). In this oxidative and pro-inflammatory milieu, NETs further promote lipid dysregulation by facilitating LDL oxidation and impairing HDL anti-oxidative and cholesterol efflux capacities, thereby enhancing foam cell formation and plaque progression (30).

Patients with SLE exhibit elevated levels of low-density neutrophils (LDNs) in peripheral blood, which further intensify these pathological processes (31). LDNs demonstrate increased spontaneous NET formation, secrete higher levels of pro-inflammatory cytokines, and impair endothelial repair by inhibiting EPC differentiation—collectively contributing to atherosclerosis (32). Concomitantly, the structural properties of NETs enable them to act as a prothrombotic scaffold, promoting platelet adhesion and activation and facilitating coagulation cascade amplification. Through this mechanism, NETs-driven inflammation integrates with thrombus formation and plaque instability (30, 33). Moreover, excessive mitochondrial ROS production in LDNs promotes to NET release, while cytoskeletal abnormalities and altered cell-surface structures enhance their adhesion and retention within the microvascular endothelium. These alterations exacerbate microvascular damage and endothelial activation, promoting atherosclerosis through both structural and functional mechanisms (34).

#### 2.2.3 Macrophages

Macrophages play a pronounced pro-atherogenic role in SLE–associated atherosclerosis. During active disease, patients exhibit elevated peripheral monocyte counts, which readily differentiate into macrophages exhibiting enhanced pro-inflammatory activity and impaired autophagic function (35). Immunoregulatory disturbances further shape macrophage behavior in SLE. IFN-I, IL-17, and aberrant CD40 signaling collectively promote macrophage polarization toward the pro-inflammatory M1 phenotype (36). M1 macrophages undergo metabolic reprogramming characterized by enhanced glycolysis and diminished oxidative phosphorylation, and they promote vascular inflammation through NF-κB, TLRs, and related pathways. These processes exacerbate vascular injury and accelerate atherosclerosis (37). In terms of lipid metabolism, macrophages in SLE mice demonstrate impaired ABCA1/ABCG1-mediated cholesterol efflux, resulting in intracellular lipid accumulation and increased foam-cell formation, contributing to lipid deposition and plaque progression (38). Furthermore, defective autophagy in macrophages accelerates atherogenesis. Autophagic impairment results in excessive inflammasome activation, lysosomal dysfunction, and accumulation of cytotoxic protein aggregates, maintaining macrophages in chronically inflamed and lipid-laden state (39). These abnormalities synergistically accelerate the initiation and growth of atherosclerosis in individuals with SLE.

#### 2.2.4 T cells and B cells

Patients with SLE exhibit significant imbalances in T- and B-lymphocyte subsets, accompanied by features of immunosenescence, which collectively underlie the early development of atherosclerosis (40). In T cells, persistent activation of calcium/calmodulin-dependent protein kinase IV promotes CREM-mediated suppression of IL-2 and a consequent reduction in regulatory T cells (Tregs). Simultaneously, activation of the JAK and PI3K/AKT/mTOR pathways enhances Th17 abundance and promotes Th17 differentiation (41). Impaired Treg function diminishes the secretion of anti-inflammatory cytokines such as IL-10 and TGF-β, failing to restrain the excessive activation of autoreactive T cells and innate immune cells (42). Elevated Th17 cells and IL-17 further amplify vascular inflammation by recruiting monocytes and macrophages, activating NF-κB, and inducing endothelial expression of VCAM-1, collectively accelerating atherogenesis (42, 43). Natural killer T (NKT) cells are reduced in number and functionally impaired in SLE patients, weakening their protective role in early atherogenesis, which normally involves IL-10 production and suppression of inflammatory activation (44). In addition, circulating angiogenic T cells in SLE show signs of senescence and cytotoxicity, impairing EPC differentiation and vascular repair capacity, which exacerbates endothelial injury and increasing cardiovascular risk (12).

Aberrant B-cell activation and subset imbalance also play critical roles in atherosclerosis progression in SLE (45). The B-cell activating factor (BAFF) receptor pathway, a canonical driver of B2-cell activation, serves as a key promoter of atherogenesis (46). Elevated BAFF levels, commonly observed in SLE, can directly induce apoptosis in endothelial cells and EPCs, thereby impairing endothelial regeneration. Excessive BAFF signaling also hyperactivates B2 cells, amplifying humoral immune responses and sustaining systemic and vascular inflammation (12). Supporting these findings, Theodorou et al. reported that SLE patients with high circulating BAFF concentrations exhibited significantly greater plaque burden and increased arterial wall thickness compared to those with lower BAFF levels (47).

#### 2.2.5 Autoantibodies

A broad spectrum of pathogenic autoantibodies is a defining immunological feature of SLE, and these antibodies participate directly or via immune complex formation in multiple pathogenic processes that accelerate atherogenesis. Antiphospholipid antibodies promote endothelial, monocytic, and complement activation, induce adhesion molecule and tissue factor expression, and enhance platelet activation, thereby significantly increasing thrombosis risk and promoting intimal hyperplasia (48). Autoantibodies targeting lipid-metabolizing enzymes or apolipoproteins can interfere with lipoprotein catabolism and clearance, exacerbating dyslipidemia. Furthermore, immune complexes formed by antiphospholipid antibodies, ox-LDL, and β2-glycoprotein I (β2GPI) trigger abnormal phagocytosis and pro-inflammatory cytokine release in macrophages, amplifying local vascular inflammation and facilitating plaque development (49). Anti-dsDNA antibodies, a serological hallmark of SLE individuals, recognize multiple autoantigens and induce inflammation, apoptosis, and tissue injury (50). These antibodies activate endothelial cells, upregulate VCAM-1 and ICAM-1 expression, and promote immune cell infiltration. They are also closely associated with increased NET formation and heightened oxidative stress, which together intensify vascular inflammation and contribute to plaque destabilization (51). Additionally, compared with healthy individuals, patients with SLE exhibit reduced titers of protective anti-atherosclerotic antibodies (e.g., anti–malondialdehyde antibody), which weakens the clearance of inflammatory and oxidative products and increases the arterial wall susceptibility to a persistent pro-inflammatory milieu (45). Collectively, lupus-associated autoantibodies not only reflect systemic immune activation but actively participate in vascular injury, lipid modification, and thrombosis, thereby bridging systemic autoimmunity and local plaque evolution.

### 2.3 Genetic and epigenetic mechanisms

Genetic susceptibility contributes to the increased burden of atherosclerosis in patients with SLE. Recent transcriptomic and genomic studies have identified several key hub genes including SPI1, MMP9, C1QA, CX3CR1, MNDA, CCR1, CD163, IL1RN, and SIGLEC1, together with clusters of differentially expressed genes. These genes are predominantly enriched in pro-inflammatory pathways such as IL-17, TNF-α, and NF-κB, and demonstrate strong correlations with immune cell infiltration. These findings suggest an inherent transcriptional predisposition toward inflammatory activation and monocyte/macrophage differentiation (52–54).

Beyond genetic risks, epigenetic mechanisms have emerged as critical regulators of these pathways. For instance, altered expression of microRNAs (miRNAs) such as miR-146a and miR-155, which are known to modulate NF-κB signaling and innate immune responses, has been linked to endothelial dysfunction and plaque vulnerability in SLE (55). Specifically, the SLE risk variant rs2431697 disrupts a distal enhancer that physically interacts with the miR-146a promoter; the protective allele facilitates NF-κB binding, increasing miR-146a expression and suppressing IFN-1 pathway activation, illustrating a direct genetic-epigenetic interplay in immune dysregulation (56). Furthermore, specific DNA methylation patterns in genes related to lipid metabolism and interferon signaling may contribute to the persistent pro-inflammatory state observed in patients with subclinical atherosclerosis (57). Genetic variants including MTHFR C677T and BAFF polymorphisms, and dysregulated mRNA levels of TNFSF4 and TRAF2 have also been linked to subclinical atherosclerosis in SLE patients. These observations indicate that genetic and epigenetic mechanisms jointly shape immune dysregulation and lipid-metabolic disturbances, thereby influencing vascular risk (47, 58–60). Furthermore, macrophage autophagy-related genes, particularly ATG5 and ATG7, have been implicated in the early pathogenesis of atherosclerosis in SLE mice, underscoring the role of impaired immune-metabolic homeostasis in premature vascular injury (61, 62).

Inherited and population-level differences also contribute to cardiovascular risk disparities across racial and ethnic groups, which may act in parallel with genetic and epigenetic pathways. Large population-based studies have identified Black individuals as a high-risk group for adverse cardiovascular outcomes including stroke, heart failure, and overall mortality in both SLE and lupus nephritis-related end-stage renal disease, whereas Hispanic and Asian populations generally exhibit lower myocardial infarction risk and more favorable survival (63, 64). However, these disparities have important limitations and cannot be attributed solely to biologic or genetic factors; they may also be heavily influenced by differences in access to healthcare, socioeconomic status, social environmental conditions, and quality of care, which may modify the expression of underlying genetic and epigenetic risks in diverse populations. Thus, racial and ethnic differences in cardiovascular risk among SLE patients reflect a complex interplay of biological, genetic, and social determinants.

### 2.4 Pharmacologic therapies

Long-term pharmacological therapy for SLE is essential for suppressing immune-mediated inflammation and maintaining disease control. However, several commonly used therapeutic agents may adversely influence the progression of atherosclerosis. High-dose or prolonged glucocorticoid therapy induces metabolic disturbances including hyperglycemia, dyslipidemia, and elevated blood pressure, and may aggravate endothelial dysfunction, thereby increasing atherosclerotic risk (65). Certain immunosuppressants, particularly calcineurin inhibitors, have also been implicated in vascular injury. These agents activate the TLR4 signaling, upregulating adhesion molecules including ICAM-1 and VCAM-1 in endothelial and vascular smooth muscle cells, together with elevating levels of pro-inflammatory cytokines (e.g., IL-6, TNF-α, IL-1β), collectively amplifying vascular inflammation (66). In addition, calcineurin inhibitors modulate the RhoA/ROCK pathway, enhance intracellular calcium influx in smooth muscle cells, and potentiate vasoconstriction. They further elevate ROS while suppressing superoxide dismutase-2 (SOD2) activity, thereby intensifying oxidative stress, disrupting vascular homeostasis, and contributing to the heightened risk of coronary atherosclerosis in SLE patients (67).

## 3 Clinical implications

SLE–associated atherosclerosis is a homogeneous and stratified process shaped by age, sex, and racial/ethnic background. Recognizing this heterogeneity is essential for accurate cardiovascular risk assessment and targeted prevention strategies. Age-dependent differences suggest that conventional cardiovascular risk prediction models underestimate risk in younger patients with SLE. Notably, childhood- and adolescent-onset SLE is associated with a significantly higher cardiovascular risk and an earlier onset of cardiovascular events compared with adult-onset SLE and age-matched healthy controls, highlighting the profound impact of early and sustained immune dysregulation on vascular health (68, 69). Despite low absolute risk, young individuals particularly premenopausal women exhibit disproportionately elevated relative risk of atherosclerotic cardiovascular disease. This excess risk is predominantly driven by immune-mediated mechanisms rather than traditional metabolic factors (4). Early vascular injury is closely linked to IFN-I activation, NETs formation, and pro-inflammatory HDL (70, 71). Therefore, early screening strategies should also incorporate disease-specific indicators such as disease activity, autoantibody profiles, and cumulative inflammatory burden (72, 73). Adjunctive vascular imaging (e.g., carotid intima–media thickness or plaque assessment) may be particularly valuable in this population (19).

In contrast, in older patients or those with long-standing disease, the underlying mechanisms shift toward a combined immune–metabolic phenotype (9). Chronic glucocorticoid exposure, renal impairment, and cumulative organ damage contribute to insulin resistance, dyslipidemia, and endothelial dysfunction (74). These factors amplify traditional cardiovascular pathways. Accordingly, management should prioritize both tight disease control and aggressive modification of conventional risk factors, including lipid levels, blood pressure, and glucocorticoid exposure.

Sex-specific differences add another layer of complexity. The cardiovascular protection typically observed in premenopausal women is largely abolished in SLE. This paradox reflects qualitative alterations in lipoprotein function, particularly the conversion of HDL into a pro-inflammatory form, as well as interactions between sex hormones and immune activation pathways. Consequently, female patients with SLE especially younger women should be regarded as a high-risk group despite otherwise favorable traditional risk profiles (75, 76). Although less frequently affected, male patients often present with a higher burden of traditional risk factors and more severe organ damage, suggesting a distinct phenotype in which immune and metabolic mechanisms coexist (77).

Racial and ethnic disparities further influence cardiovascular outcomes. These differences arise from a complex interplay of genetic susceptibility, immunological phenotype, disease severity, and social determinants of health (78). Patients from certain racial or ethnic backgrounds may exhibit heightened interferon pathway activation, higher prevalence of antiphospholipid antibodies, and more severe disease manifestations such as lupus nephritis, all of which contribute to accelerated vascular injury (79, 80). In addition, disparities in healthcare access, treatment adherence, and therapeutic exposure may further modulate cardiovascular outcomes. These observations underscore the need for culturally and socioeconomically tailored prevention strategies.

Taken together, these findings support a precision medicine approach to cardiovascular risk management in SLE. Rather than applying a uniform strategy, clinicians should adopt a stratified framework that accounts for immunological activity, metabolic status, demographic characteristics, and treatment-related factors. Future risk models incorporating disease-specific biomarkers may improve early risk identification. Ultimately, targeted interventions addressing both immune dysregulation and cardiometabolic abnormalities may mitigate the burden of premature atherosclerosis in this complex patient population.

## 4 Discussion and conclusion

**Figure 1:**
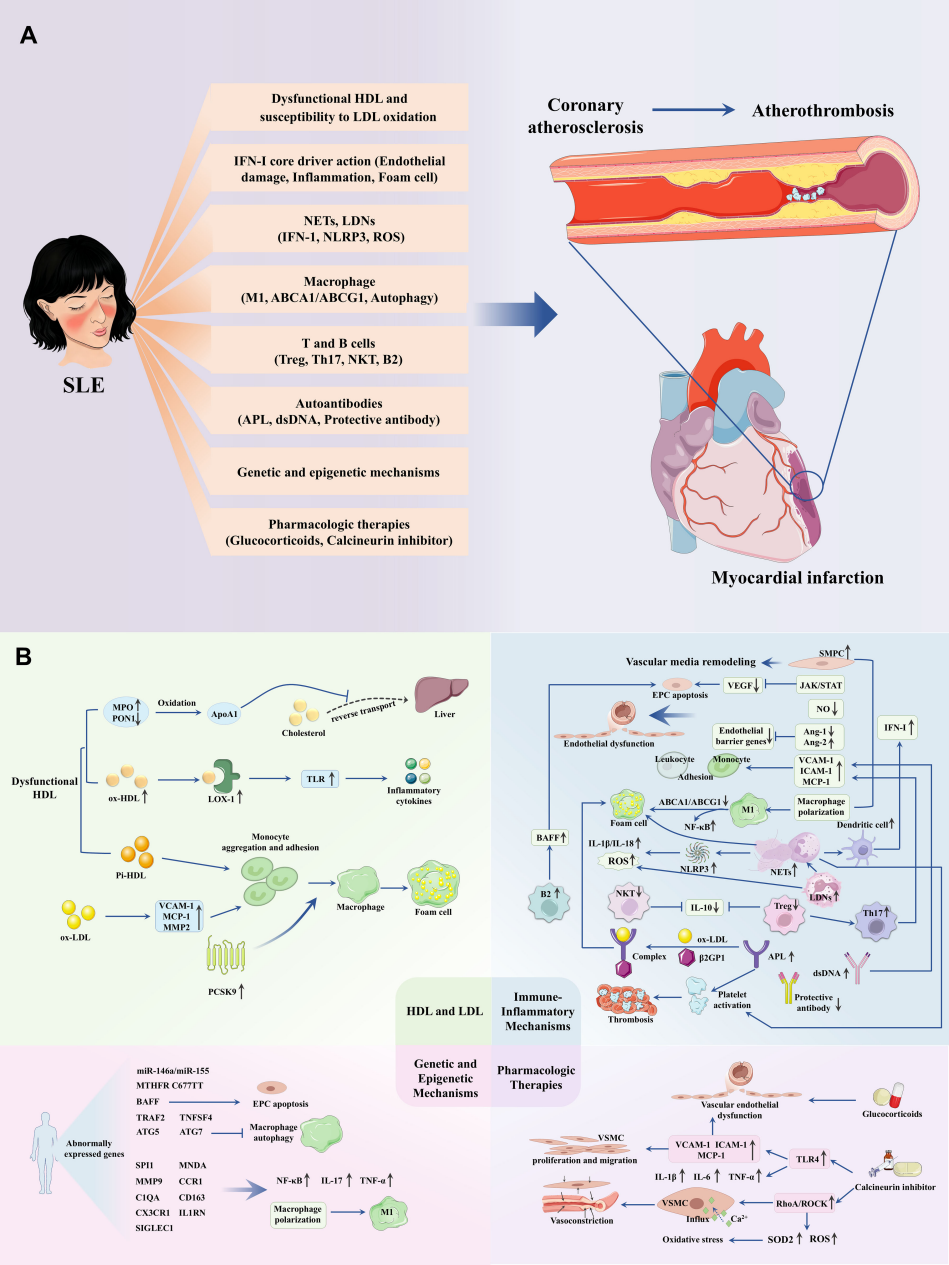
SLE accelerates coronary atherosclerosis. **(A)** Overview of the pathological processes by which SLE promotes atherosclerosis. **(B)** Detailed pathological mechanisms of SLE promoting atherosclerosis. Dysfunctional HDL, ox-LDL, IFN-I, NETs, low-density neutrophils, macrophages, T and B cells, autoantibodies (such as antiphospholipid and anti-dsDNA antibodies), as well as genetic and transcriptional alterations, together with commonly used SLE medications (e.g., glucocorticoids and calcineurin inhibitors), collectively accelerate atherosclerosis progression in SLE and increase the risk of cardiovascular events such as myocardial infarction.

SLE-associated atherosclerosis represents a prototypical model of immune-driven vascular disease, in which classical lipid-centered paradigms are insufficient to explain the magnitude and timing of cardiovascular risk. This review highlights that immune dysregulation, including IFN-I signaling, NET formation, and maladaptive innate–adaptive immune crosstalk, serves not merely as a modifier but as a central driver of atherogenesis in SLE. These processes intersect with, and often amplify, traditional metabolic pathways, resulting in a hybrid pathogenic framework that evolves over the disease course (**Figure 1**).

IFN-1 signaling, excessive NET formation, macrophage inflammatory polarization, imbalances in T and B cells, and pathogenic autoantibodies collectively promote endothelial injury, foam cell formation, and vascular remodeling. Concurrently, dysfunctional HDL and enhanced LDL oxidation amplify inflammatory signaling within the arterial wall. These mechanisms form a feed-forward loop in which inflammation modifies lipoproteins, and oxidized lipoproteins further activate immune pathways, thereby sustaining vascular injury. However, the hierarchy of these pathways are not fully delineated, and may vary across disease stages and patient subgroups. And the temporal dynamics of vascular injury in SLE are incompletely understood. It remains unclear when immune-mediated endothelial dysfunction transitions into irreversible structural atherosclerosis, and which biomarkers most accurately capture this shift. Additionally, while dysfunctional HDL and oxidized LDL are well described, their causal roles versus epiphenomenal associations require further clarification through longitudinal and interventional studies. The impact of pharmacologic therapies is also complex and sometimes paradoxical, as agents that suppress inflammation may simultaneously exacerbate metabolic risk (81).

Another major challenge lies in translating mechanistic insights into clinical benefit. The failure of statin trials underscores the limitation of targeting isolated pathways in a multifactorial disease. Emerging strategies, including modulation of IFN-I signaling, inhibition of NETs formation, and targeting of immune–metabolic interfaces, hold promise but lack robust clinical validation. Furthermore, heterogeneity across age, sex, and racial/ethnic groups suggests that uniform therapeutic strategies are unlikely to succeed, reinforcing the need for stratified and precision-based approaches to cardiovascular risk management in SLE.

In conclusion, SLE-associated atherosclerosis arises from a dynamic interplay between immune activation and metabolic dysfunction, with immune mechanisms predominating in early disease. Future research should prioritize longitudinal studies, integrated multi-omics approaches, and biomarker-driven clinical trials to better define pathogenic hierarchies and therapeutic windows. A deeper understanding of these processes will be essential for developing targeted interventions capable of reducing the burden of premature cardiovascular disease in SLE.
